# Enhanced Supersaturation via Fusion-Assisted Amorphization during FDM 3D Printing of Crystalline Poorly Soluble Drug Loaded Filaments

**DOI:** 10.3390/pharmaceutics13111857

**Published:** 2021-11-04

**Authors:** Guluzar Gorkem Buyukgoz, Christopher Gordon Kossor, Rajesh N. Davé

**Affiliations:** Otto H. York Department of Chemical and Materials Engineering, New Jersey Institute of Technology, Newark, NJ 07102, USA; gg275@njit.edu (G.G.B.); cgk6@njit.edu (C.G.K.)

**Keywords:** FDM 3D printing, amorphous solid dispersions (ASDs), tablet design, tablet surface area, drug supersaturation, release kinetics, filament quality

## Abstract

Filaments loaded with griseofulvin (GF), a model poorly water-soluble drug, were prepared and used for 3D printing via fused deposition modeling (FDM). GF was selected due to its high melting temperature, enabling lower temperature hot-melt extrusion (HME) keeping GF largely crystalline in the filaments, which could help mitigate the disadvantages of high HME processing temperatures such as filament quality, important for printability and the adverse effects of GF recrystallization on tablet properties. Novel aspects include single-step fusion-assisted ASDs generation during FDM 3D printing and examining the impact of tablet surface areas (SA) through printing multi-mini and square-pattern perforated tablets to further enhance drug supersaturation during dissolution. Kollicoat protect and hydroxypropyl cellulose were selected due to their low miscibility with GF, necessary to produce crystalline filaments. The drug solid-state was assessed via XRPD, DSC and FT-IR. At 165 °C HME processing temperature, the filaments containing ~80% crystalline GF were printable. Fusion-assisted 3D printing led to GF supersaturation of ~153% for cylindrical tablets and ~293% with the square-pattern perforated tablets, indicating strong monotonous impact of tablet SA. Dissolution kinetics of drug release profiles indicated Fickian transport for tablets with higher SA, demonstrating greater SA-induced drug supersaturation for well-designed 3D printed tablets.

## 1. Introduction

An increasing number of new drug entities being discovered are poorly water-soluble [1,2] which result in poor bioavailability such as the Active Pharmaceutical Ingredients (APIs) [3] Biopharmaceutic Classification System (BCS) class II drugs. One of the most successful and common strategies to address solubility limited bioavailability is the formation of amorphous solid dispersions (ASDs). This approach relies on the dispersion of a drug within hydrophilic carriers leading to an amorphous mixture [4,5,6,7,8,9,10,11,12] that potentially enhances the dissolution rate since kinetic solubility of the amorphous drug is greater than the crystalline counterpart [13,14]. Amongst several technologies to produce ASDs [11,13,15,16,17,18], hot-melt extrusion (HME) is a widely used method as it offers one-step, continuous and solvent-free manufacturing [6,18,19,20,21,22] and has been a method of choice for use in fused deposition modeling (FDM)-based three-dimensional (3D) printing [23,24,25,26,27]. 

FDM-based 3D printing has become a popular drug delivery platform owing to its ability to produce on-demand flexible dosages. In the FDM technique, a feed-material, usually a filament produced by HME, is fed into a heated nozzle and deposited layer by layer to form a 3D printed dosage form or device [23,24,25,28,29,30,31,32,33]. Previous studies have examined the influence of tablet design, tablet size, infill density and polymer type on the drug dissolution rate [23,24,25,26,31,33,34]. For controlling the drug release rates, 3D structure design parameters, i.e., infill density and shell thickness, were found to be critical [23,25,34], where thicker and denser structures led to slower drug release. In addition, filament preparation is an important step in FDM printing and most studies have discussed the challenges involved with filaments where the drug was present in its amorphous form [26,35]. Interestingly, few studies also considered crystalline drug form within a filament prior to 3D printing, while relying on generation of the amorphous form during the printing through the transient heating effects [27,36].

For successful FDM-based 3D printing, the filaments should have uniform diameter, sufficient mechanical resilience, lack of voids and bubbles and thermal stability [26,35,37,38,39,40]. All of which are greatly impacted by several factors such as melt rheology, HME processing temperature and individual properties of drug and polymer [20,41,42]. The same factors are also critical for designing and producing high quality ASDs regardless of their intended use. Developing the formulation and processing conditions for ASDs loaded filaments becomes challenging to simultaneously satisfy the requirements imposed by high quality ASDs production and filament printability. In particular, the best choices of polymers for given drugs and processing temperatures to generate and maintain the ASDs form [25] may not lead to acceptable filament printability. For instance, Govender et al. [35] manufactured filaments using a miscible drug-polymer formulation that generated amorphous solid dispersion. Unfortunately, filament brittleness prevented automatic feeding, leading to failure of 3D printing. Further, manual filament feeding resulted in poor dispensing precision at all of the print volumes [35]. Another example is when a drug is not fully miscible with a polymer, requiring relatively high HME processing temperatures for the API to melt and form an amorphous phase [43]. Consequently, such high processing temperatures have adverse effects on the filament quality, as reported in several studies [38,40,43,44]. Hence, producing ASDs loaded filaments that are compatible with FDM 3D printing presents several challenges.

Assuming ASDs loaded filaments could be produced with adequate properties, another challenge is in maintaining their physical stability for the duration of product shelf life. The amorphous drug forms are known as being thermodynamically unstable and tend to recrystallize [14,45,46], negating the solubility advantages of ASDs [6,23,44]. For example, Wei et al. [23] discussed formulations using haloperidol and carvedilol to produce ASDs loaded feed materials. Unfortunately, the incorporation of plasticizer into the formulation with an intention to improve the melt extrusion led to recrystallization of the APIs when tested under accelerated conditions. The physical stability could only be assured for only one of these two drugs, i.e., carvedilol, but that required reducing drug-concentration to half, about 10 wt%. One approach to circumvent the stability problems with ASD drug-loaded filaments is to produce the filaments as well as the FDM printed tablets with drug in crystalline form [47]. However, doing so would negate the advantages of the ASDs loaded filaments. Fortunately, FDM’s inherent capability to provide controllable heat energy during printing could be exploited to convert the crystalline drug loaded filaments to ASD forms and, hence, achieve the enhanced dissolution properties through simultaneous achievement of supersaturation and suppression of recrystallization. For example, Chai et al. [27] utilized thermal energy generated during 3D printing to convert the drug crystals in the filaments to amorphous form in the tablets. In principle, such fusion-assisted approach may mitigate the stability problem with amorphous drug form in the filament. Unfortunately, unless the HME process is well-designed and executed, the produced filaments may have varying amounts of amorphous drug, which is likely to recrystallize in a rather uncontrolled manner [23,48,49]. That then leads to the need of controlled heat transfer for uniformly melting non-uniformly sized and distributed drug crystals [50] and achieving full ASD conversion. Thus, ideally, the best approach for numerous candidate drugs would be to prepare the filaments with the drug in its crystalline form which simultaneously tackles the problems associated with printability of the filaments and the drug stability during storage.

Considering the potential advantages in preparing filaments that contain largely crystalline form of drug i.e., >80%, the issues related to fusion-assisted amorphous conversion during printing for generating ASDs in the printed tablets and most importantly the drug supersaturation during dissolution [6,51] are examined in this paper. It is hypothesized that by avoiding any appreciable residual amorphous content during the filament preparation step, uniform distribution of controlled sized crystalline drug particles in the filaments could promote uniform heat transfer and more effective fusion-assisted conversion to ASDs during printing. The success of this approach requires the drug to have adequately high melting temperature and minimal miscibility with the polymers at the low processing temperatures through HME operation to assure maintaining drug particles in their crystalline form. At the same time, the drug needs to have adequate miscibility at higher printing temperatures. In addition to considering these aspects, the factors affecting the dissolution performance such as tablet surface area [47,52], tablet design [34,53] and dissolution testing conditions [10,14] are examined along with their impact on the degree of drug supersaturation during dissolution.

Towards those objectives, griseofulvin (GF) is selected as a model BCS class II drug that is considered fast-crystallizing [46]. Having a high melting point, GF may better retain the drug in crystalline form and allows flexibility with setting the printing temperatures. Hydroxypropyl cellulose (HPC) is used as the matrix-forming polymer while Kollicoat^®^ Protect (KP) is used to promote drug-polymer interactions at higher processing temperatures. Drug supersaturation under non-sink conditions is examined by considering the factors affecting dissolution performance such as FDM processing temperatures, tablet design options with varying surface areas including cylindrical tablets, square-pattern perforated tablets and mini-sized tablets, as well as the dissolution vessel agitator speed. Drug supersaturation behavior during dissolution for various cases is analyzed using the Korsmeyer–Peppas model to identify better design options for the 3D tablets loaded with drug ASDs. It is hoped that the proposed investigation addressing filament design as well as tablet design aspects may further promote improved designs of FDM 3D printed tablets to achieve desirable product performance.

## 2. Materials and Methods

### 2.1. Materials

Griseofulvin (GF; Letco Medical, Decatur, AL, USA) was used as the model Biopharmaceutics Classification System (BCS) class II drug. GF is a crystalline drug with a melting point (T_m_) of 220 °C [54]. It is considered a challenging drug for the development of ASDs given the fact that it rapidly crystallizes [46]. Hydroxypropyl cellulose (HPC, SL grade, Nisso America Inc., New York, NY, USA) is a semi-crystalline polymer with the glass transition temperature (T_g_) of in range −25–0 °C and T_m_ of around 170–200 °C [42,54]. It has been reported as a suitable polymer for FDM 3D printing [26,55,56] due to its ability to produce filaments with satisfactory mechanical properties. In addition, it helps enhance the wettability of hydrophobic GF particles [57]. Kollicoat^®^ Protect (KP, BASF, Tarrytown, NY, USA) is readily soluble in water and composed of polyvinyl alcohol-polyethylene glycol graft copolymer, polyvinyl alcohol and fumed silica. It has a T_m_ of 205 °C and a T_g_ of 45 °C and is known to improve protection against moisture [58]. Further, Kollicoat^®^ Protect lowers the surface tension of water i.e., surface tension is 61.6 mN/m for 0% solution and 42.3 mN/m for 15% solution [58]. An aqueous solution of sodium dodecyl sulfate (SDS) (Sigma-Aldrich, Saint Louis, MO, USA) was used as a solvent at 7.2 g/L concentration for assay testing.

### 2.2. Preparation of Feed Materials (Filaments)

The compositions of the powder blends and HME processing parameters are shown in Table 1. The blends were mixed by a high-intensity vibrational mixer (LabRAM, Resodyn Acoustic Mixers, Inc., Butte, MT, USA) at a frequency of 61 Hz with an acceleration of 75 G for 5 min. HME processing was carried out with an 11 mm diameter co-rotating twin-screw extruder (Thermo Fisher Scientific Inc., Waltham, MA, USA) with a round-shaped die having a 2 mm opening. As similar to the procedure applied by (Moseson et al. [6], several gradually increasing levels of the HME processing temperatures were utilized (see Table 1) in an attempt to prepare filaments at varying fractions of crystalline/amorphous GF. The HME processing temperatures were selected to be lower than T_m_ of GF to ensure poor drug-polymer miscibility for avoiding the temperature-induced dissolving of the drug. The HME processing conditions were denoted with letter H and processing temperature, e.g., H160 (see Table 1). The placebo filaments used H160 and GF loaded filaments utilized H165 for printing all of the GF loaded tablets.

### 2.3. Mechanical Properties of Filaments 

The mechanical properties of the filaments from varying HME processing temperatures were assessed using 3-point bend testing in the Texture Analyzer (3-point bender tester, Instron, Norwood, MA, USA). Modulus of elasticity (ME) and tensile strength (TS), stiffness and breaking force, respectively, were considered for evaluating the filament printability [26]. There were 5–6 filaments randomly selected and cut into 1 cm segments while the diameters of the filaments were recorded using a digital caliper. The samples were placed on two support pins a 4 mm distance apart. Force was applied with a constant speed of 0.5 mm/min until samples broke. From the stress–strain plot, the slope of the initial linear segment was used to calculate ME. TS was calculated using Equation (1) [59].

(1) $$\sigma_{fs}=\frac{F_{f}L}{\Pi R^{3}}$$

Here, the flexural strength (*σ_fs_*) is defined for a circular cross-section; *F_f_* is the load at fracture; *L* is the distance between support pins and *R* is the specimen radius. The diameter, TS, and ME of the filament were plotted as the function of HME processing temperatures.

### 2.4. Printing Dosage Forms 

The objective was to produce fusion-assisted ASDs using the transient thermal energy [6] due to increased drug-polymer interactions [40], leading to formation of hydrogen bonds [60] between GF and HPC-KP. Hence, to test the effect of printing temperature on the extent of ASD formation, the temperatures above and below the melting point of GF were selected to print cylindrical tablets (see Table 2). In Table 2, the letters F and P denoted FDM 3D printing conditions. For instance, F165 represented the printed tablets laden with GF at the printing temperature of 165 °C, while P165 implied the placebo tablets. There are two design options with enhanced surface area, namely, multi mini-tablets and square-pattern perforated tablets. Therefore, these two options were utilized to prepare the tablets intended for testing the influence of tablet surface area (SA) on the supersaturation performance of GF during dissolution as shown in Table 3 in terms of relative SA values, which are normalized by the SA of regular cylindrical tablet, F240. The graphical depictions of the filament (H165), regular tablet (F240) and the tablet with the highest SA (F240d), are presented in Figure S1, Supplementary Material. The tablet designs were created using Autodesk Fusion 360 Ultimate (Autodesk 3D design software), exported as STL files and, converted to X3G files using FlashPrint software (Version 4.3.0; Jinhua, Zhejiang, China) as the slicer. The FDM 3D printer (Flashforge, Creator Pro 3D, 2016, Jinhua, Zhejiang, China) used a 0.4 mm nozzle opening for printing the tablets. The following operating parameters [47] were kept constant for all the tablets in Table 2 and Table 3; printing speed, 35 mm/s; nozzle traveling speed, 80 mm/s; layer height, 0.20 mm; infill percentage, 100%. As per the software (Autodesk 3D design software), the printing times of the tablets were estimated as F240: 4 min, F240 b: 1 min and F240 d: 18 min.

### 2.5. Fourier Transform Infrared (FT-IR) Spectroscopy

FT-IR analysis was performed with as-received GF powder, physical mixture (PM) and the printed tablets to assess the extent of drug-polymer interactions. An attenuated total reflectance (ATR) infrared spectra was collected using Agilent Cary 620 FT-IR (Santa Clara, CA, USA) equipped with single bounce diamond crystal and Golden Gate type ATR unit. Each spectrum was acquired with 32 scans with a resolution of 4 cm^−1^. The spectral data were reported in the range 1550–1750 cm^−1^ wavenumber.

### 2.6. Solid State Characterization 

As-received GF, HPC and KP powders, the filaments and the printed tablets were examined via X-ray powder diffraction (XRPD) (PANalytical, Westborough, MA, USA) to measure the extent of crystalline state of GF after thermal processing in HME and FDM 3D printing. To fit the samples to the XRPD sample holder (internal diameter, ø: 16 mm, thickness, H: 2.4 mm), tablets were printed with the set dimensions of ø: 15.5 mm and H: 1.7 mm. Slightly small dimensions were printed to take an account of polymer swelling, leading to potential increases in tablet size [47,61]. For the filaments, they were milled to prepare the compacted samples suitable for the sample holder dimensions as per the instrument specifications. The samples were scanned for 2θ angle ranging from 5° to 35° (0.01° step). To determine the % crystallinity of GF in the samples, OriginPro (Version 2020b) software (Northampton, MA, USA) was used following the previously established method reported by Rahman et al. [62]. Further, to examine the physical transformations of the drug during printing, the raw samples and the printed tablets were tested using differential scanning calorimeter (DSC 6000, Perkin Elmer, Inc., Waltham, MA, USA). In a standard aluminum pan, 5–8 mg of the sample was heated from 25 to 300 °C at a rate of 10 °C/min. Nitrogen with a flow rate of 20 mL/min was used as a purge gas.

### 2.7. Morphology 

The morphology of the particles within as-received GF and KP powders, filament (H165, Table 1) and the printed tablets (F240 and P240, Table 2) were tested via a scanning electron microscopy (JSM-7900F, JEOL Ltd., Peabody, MA, USA). The samples were placed on an aluminum stub using carbon tape and coated with gold via a sputter coater (Q150T 16017, Quorum Technologies Ltd., Laughton, East Sussex, England). The images from each sample were recorded. To further examine the morphology of the GF particles in the filament (H165, Table 1) and printed tablet (F240, Table 2), the polarized light microscopy (PLM, Axio Scope.A1, Carl Zeiss Microscopy GmbH, Göttingen, Germany) was used at 10X resolution. For better visualization, thinner tablets of ~0.1 mm thickness were printed, corresponding to cases F165 and F240. 

### 2.8. Thermal Analysis 

Thermo-gravimetric analysis (TGA) was performed using a TGA/DSC1/SF STARe system (Mettler Toledo Inc., Columbus, OH, USA) to assess the extent of thermal degradation of the compounds. Tested samples included as-received GF, HPC and KP powders, their physical mixture (PM) and the printed tablet, F240. Testing involved placing 5–8 mg sample in a standard ceramic crucible, heated from 25 to 300 °C at a rate of 10 °C/min and cooled back to 25 °C under a nitrogen flow.

### 2.9. Determination of Drug Concentration 

It was hypothesized that the drug content uniformity (CU) of the larger size tablets is almost certain if such small size tablets achieved excellent CU. Thus, for better discernment of content uniformity and reducing the need for the required solvent usage during assay testing in numerous cases, smaller tablets with the set dimensions of ø: 5 mm × H: 1 mm were considered and accordingly printed at the same printing parameters described in Section 2.4. The samples were dissolved in 150 mL of 7.2 g/L SDS solution and stirred via magnetic bars overnight. A Thermo Scientific Evolution 300 UV–vis spectrophotometer (Thermo Fisher Scientific Inc., Waltham, MA, USA) was used to measure the UV absorbance at a wavelength of 297 nm for the dissolved samples. The tablet mass and dimensions were recorded. The relative standard deviations (RSDs) in the drug concentrations were calculated for each set containing a minimum of 3 samples (*n* = minimum 3 and up to 5 in a few cases).

### 2.10. In-Vitro Dissolution, Supersaturation and Release Kinetics of GF Tablets

Printed GF tablet designs, discussed in Section 2.4, namely, cylindrical tablets, as well as multi mini-tablets and square-pattern perforated tablets were tested for their dissolution performance. Dissolution of ASDs under non-sink conditions can generate supersaturated solution which is thermodynamically metastable or unstable [63], which may give rise to nucleation, crystal growth and eventually recrystallization [63]. Thus, non-sink dissolution condition, a common characterization method for assessing the supersaturation performance and recrystallization behavior of a drug, was selected. The tablets were designed and printed with a high dose of GF (~90 mg) [64] and, hence, large size of ø: 16 mm × H: 2 mm. This could be considered nearly extreme non-sink dissolution conditions as per Sun et al. [10,51]. This moderately high supersaturation condition would enable assessment of both dissolution and recrystallization behaviors by providing robust non-sink conditions with respect to crystalline solubility [6]. Deionized (DI) water was selected as a dissolution medium as it better discriminates the GF formulation [57,65,66] and is a second commonly used dissolution medium for ASDs [14]. The dissolution paddle apparatus (USP II, Sotax, Basel, Switzerland) was used. The samples were added to 1000 mL of DI water at 37 °C. The paddle speed of 50 rpm was used for testing all the printed tablets except one case, i.e., F240a (see Table 2 and Table 3). The paddle speed for F240a testing was at 250 rpm [23,67]. The higher agitator speed was expected to give rise to a faster disintegration of the printed tablets; hence, it served as a control for testing the effect of faster increase in drug dissolution on the supersaturation behavior. The supersaturation performance of PM and H165 served as non ASD control options. Supersaturation behaviors of the tablets having enhanced tablet surface areas were also tested. Dissolution testing of the multi-mini tablet design, F240b, could not be reliably done without using the sinkers owing to their very small size. However, for the square-pattern perforated tablet design, testing was done with (F240c) and without (F240d) basket sinkers. It is noted that floating is considered to be biorelevant [28]. Aliquots were withdrawn over 24 h and filtered with 0.45 µm nylon membrane-type syringe filters (Celltreat scientific products, Pepperell, MA, USA). The filtrates were diluted with the dissolution medium. The 3–4 replicates were performed for each sample. The average amount of drug dissolved was plotted as a function of time (minute). As previously reported by Rahman et al. [64], the relative % supersaturation was calculated such that GF concentrations at ~12 h of dissolution were normalized with the thermodynamic solubility of as-received GF (14.2 mg/L).

The Korsmeyer–Peppas model, depicted in Equation (2), was employed for mechanistically analyzing the dissolution profiles of the printed tablets, Table 3, to better understand the supersaturation performance of GF. This model is commonly applied for describing the drug release behavior from dense polymeric matrices, such as FDM 3D printed tablets [28,34,47,68]. The model was fitted to dissolution profiles for the first 4 h, which represent about 60% of total GF dissolution for the case with the highest supersaturation, F240d.

(2) $$F\;=k\times t^{n}$$

where *F* is the percentage of the drug dissolved at time *t*, *k* is a constant incorporating structural and geometric characteristics of the drug dosage form and *n* is the release exponent, indicative of the drug release mechanism. The exponent represents Fickian diffusion for *n* = 0.45, Case II transport with *n* = 0.89 and, anomalous transport with 0.45 < *n* < 0.89 for cylindrical geometry while the lower and the upper limits of *n* are 0.5 and 1 for the slab geometry, respectively [69]. It is noted that all tablets are cylindrical, while the square-pattern perforated tablet designs, F240c and F240d, could be assumed to have a thick slab (film) geometry as per Alhijjaj et al. [70].

### 2.11. Stability Testing 

The stability testing was carried out for the samples H165 and F240. The samples were placed in a plastic bag and stored at room temperature (20–25 °C) and humidity (35 ± 5% RH) for one month. The samples were analyzed through dissolution tests as discussed in 2.10.

## 3. Results

### 3.1. Fabrication of Filaments

Filaments were produced by adjusting the processing parameters (Table 1) to vary the fractions of crystalline/amorphous GF. As mentioned earlier, the processing temperature was kept lower than T_m_ of GF [6] and the minimum operable temperature for successful HME processing was 165 °C. Further lowering the temperature detrimentally increased the required torque, which is not recommended for the instrument safety [23,38]. The processing temperature was gradually elevated to produce filaments containing amorphous GF. However, at 210 °C (H210, Table 1), the filament was liquid-like at the point of extrusion from the die owing to the reduced viscosity by the temperature. In addition, a substantially high rotation speed (Table 1) for the screws was required to properly convey the material through the barrel. Thus, increasing the temperature further was found ill-advised. The filaments produced at the temperature in the range of 165–190 °C (H165–H190) were opaque, while those produced at 210 °C (H210) were clearer, implying the formation of the amorphous structure [6]. The degree of GF crystallinity in the filaments is an important attribute that will be further discussed in the following sections.

### 3.2. Filaments Quality

**Figure 1:**
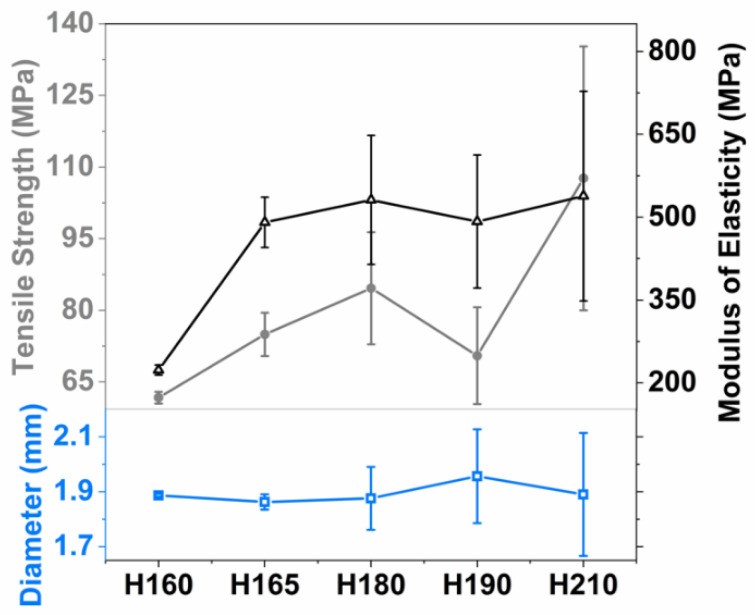
Mechanical properties and diameters of the filaments with varying HME processing temperatures, ranging 160–210 °C.

The effect of HME processing temperatures on the filament printability was assessed by analyzing their mechanical resilience, i.e., tensile strength (TS) and modulus of elasticity (ME), as well as the uniformity of filament diameters (see Figure 1). Although there are no established standards for acceptable values of mechanical properties to assure printability, attaining high breaking stress, high stiffness, long breaking distance, as well as diameter uniformity indicate better printability [26,71]. The TS and ME were the lowest for the placebo filament, H160, and increased in the presence of drug particles. The higher ME, which means increased stiffness, for the drug-loaded filaments, H165–H210, could be attributed to the polymer network being disrupted in presence of the drug [72]. As the processing temperature increased, the filaments, H165–H210, exhibited increasing TS but similar ME, which may indicate ME was influenced more by the existence of GF particles than the processing temperature. The increasing TS could be attributed to the drug-polymer interactions, where stronger interactions were expected at the higher processing temperatures due to the melting of the drug, which potentially forms more homogenous matrix. This outcome is in line with Yang et al. [38], where a similar trend of increasing TS with increasing temperature was reported. Further, as the processing temperature increased, the standard deviations in diameters of the filaments were considerably increased, with H210 being the largest (see Figure 1). Therefore, H210 was not printable even when it, like all others, possessed satisfactorily high mechanical properties. The lack of uniformity in the diameter was due to its propensity to spread out upon exit from the die. It was either too thick for the transfer column through heater or too thin for the conduction rolls inside the print head of the 3D printer. Such poor quality for the filaments associated with high processing temperatures has been previously reported, which included dispensing imprecision, dosing inaccuracy, or poor mechanical resilience [35,38,40]. Consequently, H165 was selected for printing the tablets due to its satisfactory mechanical resilience and highly uniform diameter. Such results demonstrate the challenges in producing ASD loaded filaments that also have satisfactory filament quality necessary for printing.

### 3.3. FT-IR Spectroscopy

**Figure 2:**
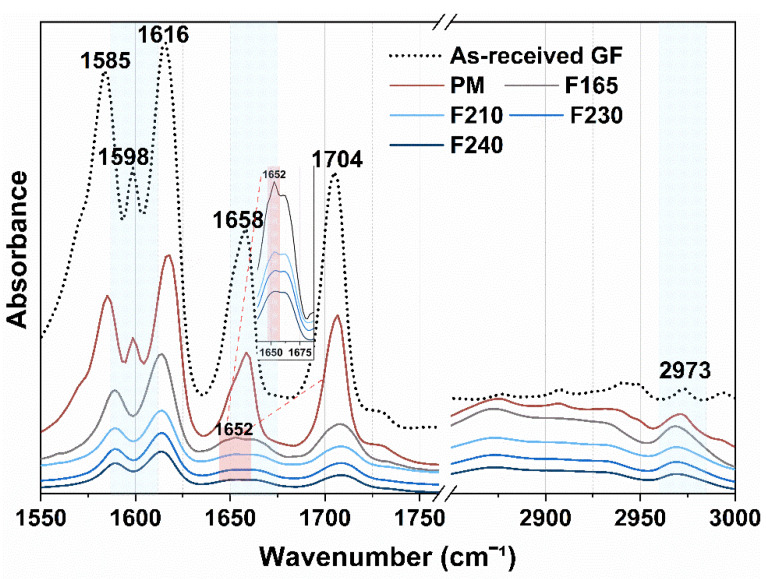
FT-IR spectra of as-received GF, physical mixture (PM) and 3D printed tablets through F165-F240.

The FT-IR spectra of as-received GF, PM and printed tablets are presented in Figure 2 to assess molecular interactions, if any, between the drug and polymers. The FT-IR spectrum of as-received GF exhibited characteristic peaks in the region 1550–1800 cm^−1^ corresponding to the C=O stretching vibrational frequencies, which are in line with Bennett et al. and Yadav et al. [73,74]. It is expected that the hydroxyl groups of HPC and KP can potentially form hydrogen bonds with the carbonyl groups of GF [54,75]. The IR spectra indicating hydrogen bonding often contain peaks that are shifted, broadened, or had lower intensity, signaling the formation of amorphous structure and strong drug-polymer interactions [54,60]. As seen in Figure 2, the skeleton stretches of the PM and as-received GF had similarity while the PM had inappreciably lower intensity, indicating no evidence of strong molecular interactions [76]. For H165, the peak at 1606 cm^−1^ no longer existed and the neighboring peaks broadened and shifted. In addition, the peak at 1658^−1^, C=O stretching, broadened and split into two sub-peaks of 1652 and 1662 cm^−1^. The broadening refers to the distribution of free and bound carbonyl groups of GF [73,77]. This may suggest a disruption in drug-drug interactions in favor of drug-polymer interactions, most likely due to the increased mobility in chains during melt extrusion [32,40]. As the processing temperature increased through F210–F240, the peak intensities decreased. Particularly, the peak at 1652 cm^−1^ monotonically decreased and eventually diminished in the spectrum of F240. Similarly, as the processing temperature increased, examination of the C–H stretching in 2800 to 3150 cm^−1^ range [73,76], exhibited peaks that are both decreased in intensity and shifted from 2973 to 2969 cm^−1^ (see Figure 2). This could be attributed to the lack of long-range order and enhanced hydrogen-bonding interactions [73,78]. That suggested hydrogen bonding was proportional to the increasing processing temperatures through 210–240 °C, being strongest at 240 °C. As hydrogen bonds refer to the amorphization, these outcomes imply that the degree of amorphization in the printed tablets was in the order of F240 > F230 > F210.

### 3.4. Crystallinity

**Figure 3:**
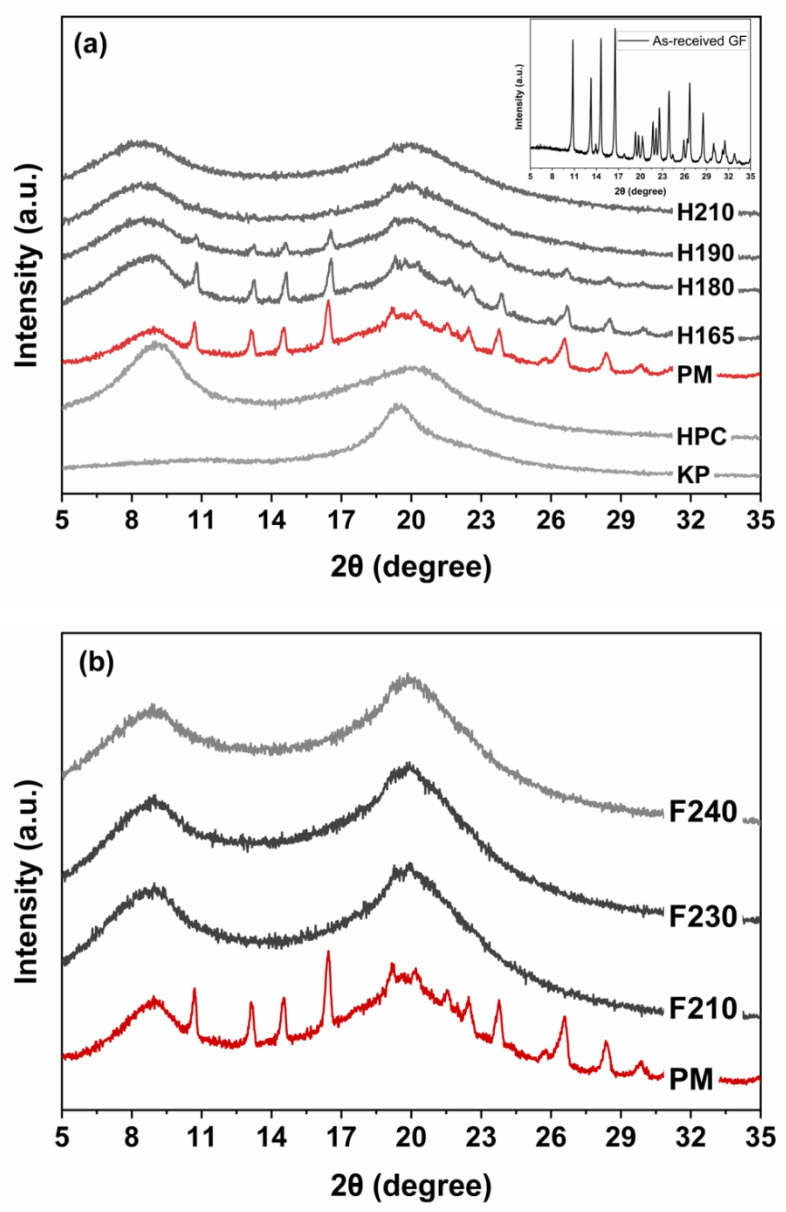
XRPD pattern of (**a**) as-received GF, physical mixture (PM), filaments through H165-210 and (**b**) 3D printed tablets, F210-F240.

The PM, filaments and printed tablets were examined using XRPD and DSC to identify any solid-state changes to GF after thermal processing in HME and FDM of as-received GF, HPC and KP. The XRPD diffractograms were presented in Figure 3. As-received GF showed characteristic peaks [62], whereas polymers exhibited a halo pattern indicating their either amorphous nature or non-detectable amount of crystallinity, Figure 3a. The PM showed similar characteristic peaks, yet with lower intensity (see Figure S2, Supplementary Material for XRPD diffractograms of the GF and PM with the same scale range). This is expected due to the surface coverage and dilution of GF particles with the polymers [54,57]. The diffractograms of H165 and PM resembled along with their comparable peak intensities. The % crystallinity of GF in H165 was estimated to be 83.06%. That confirmed that GF particles mostly distributed [79] within the polymeric matrix and remained largely crystalline. The reduced % crystallinity of GF, approximately ~17%, for H165 could be explained with the formation of amorphous GF stemming from temperature-induced drug-polymer miscibility [79] (see Section 3.3). Further increasing the processing temperature in HME further lowered the peak intensities of H180 and H190, indicating a further reduction in % crystallinity of GF. Eventually, H210 showed a halo pattern, as the characteristic peaks of GF disappeared. Therefore, at 210 °C, ASDs loaded filament was produced. A similar observation was valid for FDM printed tablets F210 through F240, as all showed a halo pattern (see Figure 3b). These halo patterns confirmed that largely crystalline GF in the filament was successfully transferred to ASDs by fusion-assisted melting via FDM 3D printing at the temperature range of 210–240 °C. These outcomes were further supported by the DSC results, presented in the supplementary material to conserve space (see Figure S3, Supplementary Material).

### 3.5. SEM Images

**Figure 4:**
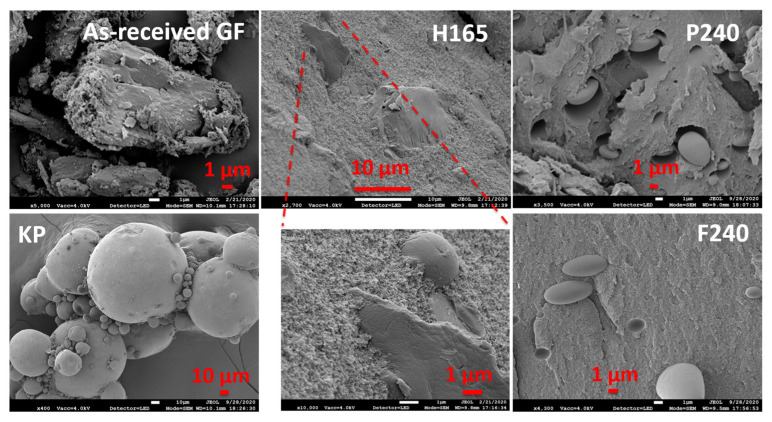
SEM images of as-received GF, KP powders, filament (H165), 3D printed tablet (F240) along with 3D printed placebo tablet (P240).

The SEM images of as-received GF and KP powders, the filament (H165) and printed tablets (P240 and F240) are presented in Figure 4 to assess the morphology of the particles after thermal processing in the HME and FDM. The as-received GF had an irregular shape while KP was spherical. The SEM image and enlarged view of H165 (Figure 4) also exhibit similar morphology of what appears to be GF (irregular) and KP (spherical) embedded particles in the matrix of H165. It is noted that the particles with irregular shapes did not appear in the cross-sections of printed placebo and drug-loaded tablets, P240 and F240, while spherical particles were observed in both, as GF would have melted in those cases at the processing temperature of 240 °C during 3D printing while KP particles remained intact to some extent. These outcomes are supported by the optical microscopy images (Figure S4, Supplementary Material).

### 3.6. Thermal Stability

**Figure 5:**
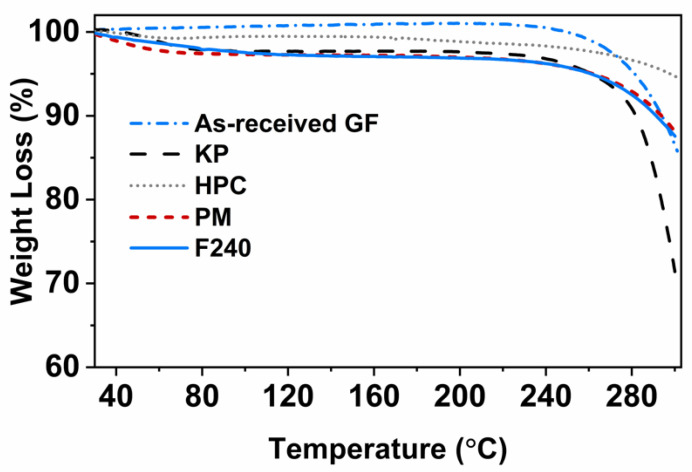
TGA thermograms of as-received GF, KP and HPC powders along with 3D printed tablet (F240).

The results for the thermo-gravimetric analysis (TGA) are presented in Figure 5. The weight loss for all the tested samples was less than 2.7% at 100 °C, which could be attributed to the free or bound water [23,80]. Similarly, Wei et al. [23] reported that 2–5% weight loss referred to the loosely bound moisture at temperatures up to 100 °C. At 240 °C, corresponding to the highest FDM processing temperature, the highest weight loss, approximately 3.8%, was seen for the PM. Since no additional weight loss was observed for the printed tablet, F240, it could be attributed to the loss of loosely bound moisture during thermal processing rather than thermal degradation, which is in line with Goyanes et al. and Wei et al. [23,61]. Therefore, no thermal degradation was observed for the printed tablets in the present study.

### 3.7. Content Uniformity of Printed Tablets

The variation (RSDs) in the tablet mass, thickness, diameter and drug concentration for the printed tablets are presented in Table 4. The RSD values in drug concentrations for all the tablets (F210–F240) were less than 1%, which corresponds to pharmaceutically acceptable content uniformity. Further, the tablet mass and dimensions had RSD values of less than 5.5%, indicating printing successfully produced accepted tablets at the printing temperatures 210 through 240 °C.

### 3.8. Drug Supersaturation and Dissolution Kinetics

#### 3.8.1. Effect of Printing Temperature

**Figure 6:**
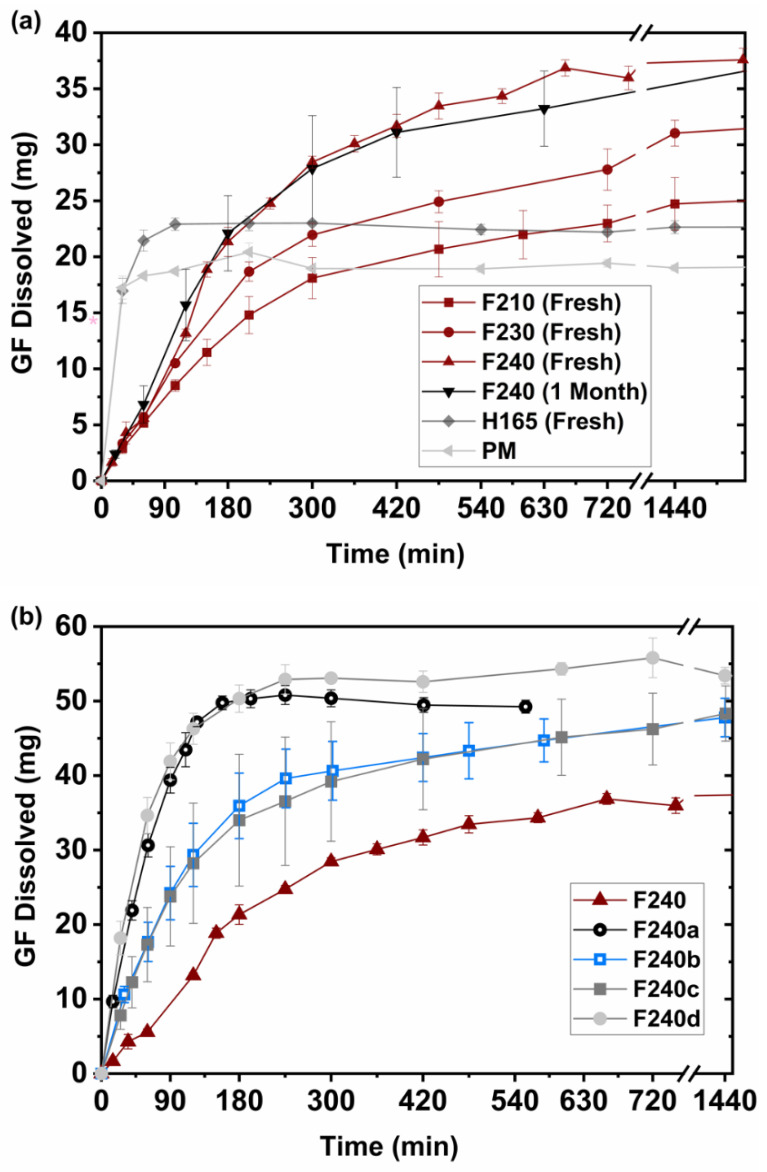
Dissolution profiles of (**a**) physical mixture (PM), filament (H165) and 3D printed tablets with varying FDM processing temperatures (F210–F240) and (**b**) 3D printed tablets with varying tablet surface area (F240b–d), as well as with the control options (F240 and F240a).

The drug dissolution profiles of all the printed tablets, discussed in Section 2.4, were tested under non-sink dissolution conditions and are presented in Figure 6. As a control, the physical mixture (PM) led to the lowest level of released GF. However, the mere presence of HPC-KP polymers enhanced the extent of GF released with respect to the thermodynamic solubility of GF (14.2 mg/L) [64]. The enhancement is likely due to the dissolution of the water-soluble polymers in the dissolution medium, which is in line with the previous reports [64,81]. The H165 and PM showed comparable dissolution rates and extent of GF released, with H165 being slightly higher. This could be attributed in part by the presence of small fraction of amorphous GF in H165 (refer to Section 3.3 and Section 3.4), which has higher kinetic solubility than its crystalline counterpart and partly by deagglomeration of as-received GF particles into polymer matrix due to HME processing. Deaaglomeration of GF seems to also evident in the content uniformity results (Table 4). For 3D printed tablets (Figure 6a), several trends were observed: (i) Initial GF release was slower for F210–F240 as compared to PM and H165, which is typical for most FDM 3D printed tablets because they are inherently dense and require longer time for drug dissolution [26,28,82]. (ii) There was no desupersaturation of GF, likely due to the gradual GF release from the matrix having water-soluble polymers, acting as crystallization inhibitors. (iii) All F210–F240 printed tablets had a higher extent of GF supersaturation compared to H165 and PM (also see Table S1, Supplementary Material) because of fusion-assisted ASDs formation, which increased with the printing temperature due to higher drug-polymer interactions (refer to Section 3.3) implying an increasing amount of amorphization (amorphous fraction of GF); F240 being the highest (153%). In addition, it is likely that the incorporation of 10 wt% KP into the formulation forged strong bonds via drug-polymer interactions, provided high miscibility with GF-HPC and reduced surface tension [58]. F240, after being stored over one month at the ambient conditions, had comparable drug release performance to the freshly prepared F240, Figure 6a, indicating the presence of strong drug–polymer interactions.

#### 3.8.2. Effects of Tablet Designs and Agitator Speed

Next, the impact of increased relative surface area (SA) for various tablet design options was examined. The tablet masses of the tested samples and their corresponding drug doses could be found in Table S2, Supplementary Material. The two designs, F240b and F240c (see Table 3), generated similar profiles (Figure 6b) and about the same level of GF supersaturation, ~215–226%, despite significantly different relative SA values (Table 3), suggesting the effect of using sinker that slowed down the water imbibition into F240c tablets. When the sinker was not used (case F240d) for the same tablet design as F240c, a further increase in GF supersaturation (293%) was achieved. The increase is likely due to increased mass-transfer because of higher available surface area that could have promoted faster water imbibition into ASDs, accelerating the drug release from the matrix. As an important outcome of the impact of tablet design, the level of GF supersaturation of these tablets (F240, F240b and F240d) monotonously increased with respect to the relative surface areas (see Figure 6b and Figure S5, Supplementary Material). Another key observation regarding the perforated tablet design option tested without a sinker (F240d), probably more bio-relevant [28], was that no evidence of GF recrystallization was seen.

As an alternate to increased SA for tablet F240, faster agitator speed of 250 rpm was used to promote increased mass transfer and faster tablet disintegration. For that experiment, termed case F240a, higher GF supersaturation to ~250% was achieved (Figure 6b). Interestingly, speeding up the dissolution rate in this manner may have resulted in a small extent of GF desupersaturation within 4h. This result may be in line with previous work indicating that a faster rise in the dissolution rate for an amorphous drug would inevitably lead to a higher maximum supersaturation, but could also lead to a sharper drop during the desupersaturation phase [83,84]. In contrast, a slower dissolution rate could avoid a sudden surge of supersaturation resulting in slower recrystallization, but lower maximum supersaturation [83,84]. While the results from F240d may support such prior observation, further work would be required to better understand the potential advantages of improved tablet design to achieve higher SA values while avoiding recrystallization. 

As per bootstrap similarity (ƒ2) test [85,86], the majority of the dissolution profiles in Figure 6b were dissimilar to each other. However, as could be anticipated from the closeness of their dissolution profiles in Figure 6b, F240a-F240d (ƒ2 = 65.15) and F240b–F240c (ƒ2 = 63.80), were similar to each other. It is notable that F240a required higher rpm during testing to achieve its performance close to F240d, whereas F240c was lower due to the sinker and just happened to provide similar dissolution profile as F240b. 

#### 3.8.3. Release Kinetics

**Figure 7:**
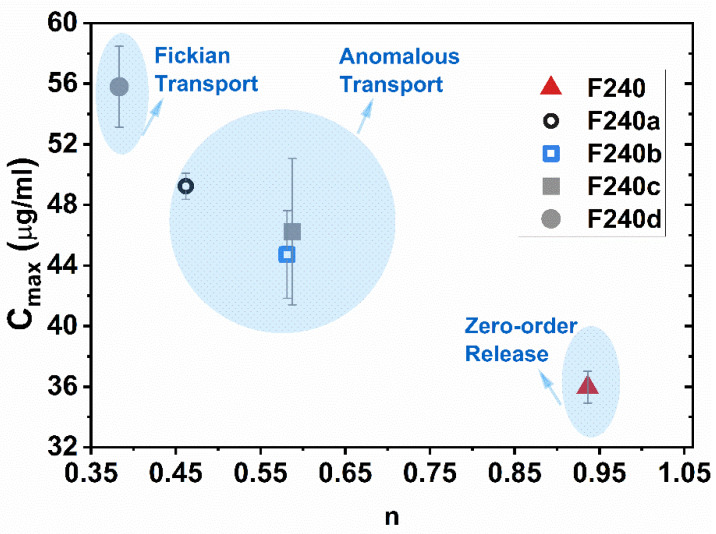
The maximum GF supersaturating concentration for the drug release from FDM 3D tablets within 12 h, as a function of the release exponent, *n*, of Equation (2).

The release kinetics of GF dissolution data were analyzed to mechanistically analyze the supersaturation performance of various designs by employing the Korsmeyer–Peppas model, Equation (2). Please refer to Table S1, Supplementary Material, to view the details of the fitting parameters including the R^2^ values. The results (see Figure 7) that show C_max_ and release exponent, *n*, suggested three different release mechanisms based on the exponent values for relevant geometry (refer to Section 2.10): (i) zero-order for F240, (ii) anomalous release transport for F240a–c and (iii) Fickian diffusion for F240d. In conjunction with the level of GF supersaturation and C_max_, Fickian transport appeared to be the most pharmaceutically relevant where the high extent of supersaturation was sustainable. The presence of dissolved polymers might have acted as crystallization inhibitors in the dissolution media, promoting the higher, sustained GF supersaturation during Fickian transport. These outcomes are in line with Sun et al. [83], where diffusion-controlled release mechanism represented the gradually increasing, yet sustained supersaturation. Nonetheless, further investigation would be required to better understand the confounding effects of the apparatus type and speed, dissolution pH and medium [51,84] as well as the extent of printed tablet density, on dissolution and supersaturation behavior.

## 4. Conclusions

The proposed methodology of lower HME processing temperature to produce filaments containing largely crystalline GF, followed by single-step fusion-assisted ASDs generation during FDM 3D printing, could achieve GF supersaturation during in vitro dissolution. This strategy significantly minimized the disadvantages of high HME processing temperatures on filament quality, important for printability and the confounding effects from GF recrystallization on tablet properties. Most importantly, avoiding large amounts of amorphous GF in the filaments led not only to improved filament diametric uniformity, but also facilitated more uniform conversion of the drug within the filaments to its amorphous form during 3D printing despite the low process shear. As a major contribution, this work showed that the tablet designs that lead to higher relative surface area (SA) could achieve higher levels of drug supersaturation. Specifically, while GF supersaturation of ~153% was possible for cylindrical tablets, with the square-pattern perforated tablets, it was nearly doubled to ~293%, indicating strong monotonous impact of tablet SA. Dissolution kinetics of drug release profiles were examined via Korsmeyer–Peppas model indicated Fickian transport for tablets with higher SA. Overall, the proposed innovative approach highlights the importance of carefully considering both the feed material processibility and printability as well as generation of single-step fusion-assisted ASDs that exploit higher SA tablet designs for achieving high drug supersaturation.

## Figures and Tables

**Table 1:** Composition of powder blends and their processing parameters in hot-melt extrusion (HME).

	Blend Composition		Processing Parameters in HME			Final Product
Run	Drug (Wt%)	Polymer (s) (Wt%)	Temperature (°C)	Screw Speed (Rpm)	Feed Rate (g/min)	
H160	-	Placebo	160	30	0.7	Filament
H165	15% GF	75% HPC + 10% KP	165	35	1.1	
H180			180	50	1.8	
H190			190	60	2.4	
H210			210	250	3.0	

“H” implies HME processing. Placebo composed of 7.5:1.0 HPC: KP.

**Table 2:** FDM 3D processing temperatures for GF-loaded and placebo tablets.

Run	Filament Used for Printing	FDM Printing Temperature (°C)	Final Product
F165	H-165	165	FDM 3D Printed Tablet
F210		210	
F230		230	
F240		240	
P165	H-160	165	
P240		240	

“F” implies FDM 3D processing; “P” implies FDM 3D printed placebo tablets. The set tablet dimensions for all the printed tablets: ø: 16 mm and H: 2 mm.

**Table 3:** Dimensions and relative surface areas for various 3D tablet designs, number of samples per dissolution vessel along with the agitator speed.

Run	3D Printed Tablets				Agitator Speed
	Design	Size ^1^	Relative SA ^2^	Quantity	(Rpm)
F240	Cylinder	16 × 2	1.0	1	50
F240a	Cylinder	16 × 2	1.0	1	250
F240b	Multi-mini w sinker	3 × 2	2.2	33	50
F240c	Structured w sinker	16 × 16 × 4.2	7.9	1	50
F240d	Structured w/o sinker	16 × 16 × 4.2	7.9	1	50

^1^ The units for the tablet size: Cylindrical geometry: mm × mm; slab geometry: mm × mm × mm. ^2^ Relative surface areas are dimensionless, as they are normalized by the SA of the regular cylindrical tablet, F240 (502.66 mm^2^).

**Table 4:** Post-printing dimensions and drug content uniformity of the FDM 3D printed tablets.

Run	Diameter (mm)	RSD	Height (mm)	RSD	Tablet Weight (mg)	RSD	DC ^1^ (%)	RSD
F165	5.57	1.11	1.29	3.31	26.46	1.33	15.79	0.33
F210	5.68	1.07	1.21	2.66	30.33	3.43	15.98	0.32
F230	5.47	0.95	1.60	1.44	34.73	1.09	15.66	0.64
F240	5.47	2.25	1.56	5.42	33.86	5.36	15.81	0.32

^1^ DC: Drug concentration.

## Data Availability

Additional data related to the paper are available and may be requested from the corresponding author.

## Supplementary Materials

The following are available online at https://www.mdpi.com/article/10.3390/pharmaceutics13111857/s1, Figure S1: Digital images of the filament, H165 (left) and FDM 3D printed tablets for cases F240 (middle) and F240d (right), Figure S2: XRPD pattern of as-received GF and physical mixture (PM), Figure S3: DSC thermograms of as-received GF, physical mixture (PM), placebo (P165 and P240) and GF loaded (F165–F240) printed tablets, Figure S4: Microscopy images of 3D tablets printed at 165 °C and 240 °C, Table S1: Extent of GF supersaturation for various tablet designs along with the fitting parameters of the corresponding fitting dissolution curves, Table S2: 3D printed tablets along with their corresponding tablet masses and theoretical drug amounts, Figure S5. Relative surface areas (SA) of 3D tablets (F240, F240b and F240d) and corresponding level of GF supersaturation. The trendline depicts monotonously increasing % GF supersaturation as a function of relative SA.

## References

[1] Gala U.H., Miller D.A., Williams III R.O. (2020). Harnessing the therapeutic potential of anticancer drugs through amorphous solid dispersions. Biochim. Et Biophys. Acta (BBA) Rev. Cancer.

[2] Lipinski C. (2002). Poor aqueous solubility—an industry wide problem in drug discovery. Am. Pharm. Rev..

[3] Amidon G.L., Lennernäs H., Shah V.P., Crison J.R. (1995). A theoretical basis for a biopharmaceutic drug classification: The correlation of in vitro drug product dissolution and in vivo bioavailability. Pharm. Res..

[4] Vasconcelos T., Sarmento B., Costa P. (2007). Solid dispersions as strategy to improve oral bioavailability of poor water soluble drugs. Drug Discov. Today.

[5] Rumondor A.C., Ivanisevic I., Bates S., Alonzo D.E., Taylor L.S. (2009). Evaluation of drug-polymer miscibility in amorphous solid dispersion systems. Pharm. Res..

[6] Moseson D.E., Parker A.S., Beaudoin S.P., Taylor L.S. (2020). Amorphous solid dispersions containing residual crystallinity: Influence of seed properties and polymer adsorption on dissolution performance. Eur. J. Pharm. Sci..

[7] Van den Mooter G. (2012). The use of amorphous solid dispersions: A formulation strategy to overcome poor solubility and dissolution rate. Drug Discov. Today Technol..

[8] Leuner C., Dressman J. (2000). Improving drug solubility for oral delivery using solid dispersions. Eur. J. Pharm. Biopharm..

[9] Lakshman J.P., Cao Y., Kowalski J., Serajuddin A.T. (2008). Application of melt extrusion in the development of a physically and chemically stable high-energy amorphous solid dispersion of a poorly water-soluble drug. Mol. Pharm..

[10] Sun D.D., Ju T.-c.R., Lee P.I. (2012). Enhanced kinetic solubility profiles of indomethacin amorphous solid dispersions in poly (2-hydroxyethyl methacrylate) hydrogels. Eur. J. Pharm. Biopharm..

[11] Hancock B.C., Zografi G. (1997). Characteristics and significance of the amorphous state in pharmaceutical systems. J. Pharm. Sci..

[12] Chiou W.L., Riegelman S. (1971). Pharmaceutical applications of solid dispersion systems. J. Pharm. Sci..

[13] Serajuddin A.T. (1999). Solid dispersion of poorly water-soluble drugs: Early promises, subsequent problems, and recent breakthroughs. J. Pharm. Sci..

[14] Newman A., Knipp G., Zografi G. (2012). Assessing the performance of amorphous solid dispersions. J. Pharm. Sci..

[15] Janssens S., Van den Mooter G. (2009). Physical chemistry of solid dispersions. J. Pharm. Pharmacol..

[16] Yu L. (2001). Amorphous pharmaceutical solids: Preparation, characterization and stabilization. Adv. Drug Deliv. Rev..

[17] Paradkar A., Ambike A.A., Jadhav B.K., Mahadik K. (2004). Characterization of curcumin–PVP solid dispersion obtained by spray drying. Int. J. Pharm..

[18] Mendonsa N., Almutairy B., Kallakunta V.R., Sarabu S., Thipsay P., Bandari S., Repka M.A. (2020). Manufacturing strategies to develop amorphous solid dispersions: An overview. J. Drug Deliv. Sci. Technol..

[19] Sarode A.L., Sandhu H., Shah N., Malick W., Zia H. (2013). Hot melt extrusion for amorphous solid dispersions: Temperature and moisture activated drug–polymer interactions for enhanced stability. Mol. Pharm..

[20] Solanki N., Gupta S.S., Serajuddin A.T. (2018). Rheological analysis of itraconazole-polymer mixtures to determine optimal melt extrusion temperature for development of amorphous solid dispersion. Eur. J. Pharm. Sci..

[21] Baghel S., Cathcart H., O’Reilly N.J. (2016). Polymeric amorphous solid dispersions: A review of amorphization, crystallization, stabilization, solid-state characterization, and aqueous solubilization of biopharmaceutical classification system class II drugs. J. Pharm. Sci..

[22] Maniruzzaman M. (2019). Pharmaceutical applications of hot-melt extrusion: Continuous manufacturing, twin-screw granulations, and 3D printing. Pharmaceutics.

[23] Wei C., Solanki N.G., Vasoya J.M., Shah A.V., Serajuddin A.T. (2020). Development of 3D Printed Tablets by Fused Deposition Modeling Using Polyvinyl Alcohol as Polymeric Matrix for Rapid Drug Release. J. Pharm. Sci..

[24] Arafat B., Qinna N., Cieszynska M., Forbes R.T., Alhnan M.A. (2018). Tailored on demand anti-coagulant dosing: An in vitro and in vivo evaluation of 3D printed purpose-designed oral dosage forms. Eur. J. Pharm. Biopharm..

[25] Solanki N.G., Tahsin M., Shah A.V., Serajuddin A.T. (2018). Formulation of 3D printed tablet for rapid drug release by fused deposition modeling: Screening polymers for drug release, drug-polymer miscibility and printability. J. Pharm. Sci..

[26] Zhang J., Feng X., Patil H., Tiwari R.V., Repka M.A. (2017). Coupling 3D printing with hot-melt extrusion to produce controlled-release tablets. Int. J. Pharm..

[27] Chai X., Chai H., Wang X., Yang J., Li J., Zhao Y., Cai W., Tao T., Xiang X. (2017). Fused deposition modeling (FDM) 3D printed tablets for intragastric floating delivery of domperidone. Sci. Rep..

[28] Ilyés K., Balogh A., Casian T., Igricz T., Borbás E., Démuth B., Vass P., Menyhárt L., Kovács N.K., Marosi G. (2019). 3D floating tablets: Appropriate 3D design from the perspective of different in vitro dissolution testing methodologies. Int. J. Pharm..

[29] Gioumouxouzis C.I., Baklavaridis A., Katsamenis O.L., Markopoulou C.K., Bouropoulos N., Tzetzis D., Fatouros D.G. (2018). A 3D printed bilayer oral solid dosage form combining metformin for prolonged and glimepiride for immediate drug delivery. Eur. J. Pharm. Sci..

[30] Jamróz W., Kurek M., Łyszczarz E., Szafraniec J., Knapik-Kowalczuk J., Syrek K., Paluch M., Jachowicz R. (2017). 3D printed orodispersible films with Aripiprazole. Int. J. Pharm..

[31] Palekar S., Nukala P.K., Mishra S.M., Kipping T., Patel K. (2019). Application of 3D printing technology and quality by design approach for development of age-appropriate pediatric formulation of baclofen. Int. J. Pharm..

[32] Scoutaris N., Ross S.A., Douroumis D. (2018). 3D printed “Starmix” drug loaded dosage forms for paediatric applications. Pharm. Res..

[33] Kempin W., Domsta V., Brecht I., Semmling B., Tillmann S., Weitschies W., Seidlitz A. (2018). Development of a dual extrusion printing technique for an acid-and thermo-labile drug. Eur. J. Pharm. Sci..

[34] Zhang J., Yang W., Vo A.Q., Feng X., Ye X., Kim D.W., Repka M.A. (2017). Hydroxypropyl methylcellulose-based controlled release dosage by melt extrusion and 3D printing: Structure and drug release correlation. Carbohydr. Polym..

[35] Govender R., Abrahmsén-Alami S., Folestad S., Larsson A. (2020). High Content Solid Dispersions for Dose Window Extension: A Basis for Design Flexibility in Fused Deposition Modelling. Pharm. Res..

[36] Jamróz W., Kurek M., Czech A., Szafraniec J., Gawlak K., Jachowicz R. (2018). 3D printing of tablets containing amorphous aripiprazole by filaments co-extrusion. Eur. J. Pharm. Biopharm..

[37] Nasereddin J.M., Wellner N., Alhijjaj M., Belton P., Qi S. (2018). Development of a simple mechanical screening method for predicting the feedability of a pharmaceutical FDM 3D printing filament. Pharm. Res..

[38] Yang Y., Chen Y., Wei Y., Li Y. (2016). 3D printing of shape memory polymer for functional part fabrication. Int. J. Adv. Manuf. Technol..

[39] Isreb A., Baj K., Wojsz M., Isreb M., Peak M., Alhnan M.A. (2019). 3D printed oral theophylline doses with innovative ‘radiator-like’design: Impact of polyethylene oxide (PEO) molecular weight. Int. J. Pharm..

[40] Kempin W., Franz C., Koster L.-C., Schneider F., Bogdahn M., Weitschies W., Seidlitz A. (2017). Assessment of different polymers and drug loads for fused deposition modeling of drug loaded implants. Eur. J. Pharm. Biopharm..

[41] Solanki N.G., Lam K., Tahsin M., Gumaste S.G., Shah A.V., Serajuddin A.T. (2019). Effects of surfactants on itraconazole-HPMCAS solid dispersion prepared by hot-melt extrusion I: Miscibility and drug release. J. Pharm. Sci..

[42] Sarode A., Wang P., Cote C., Worthen D.R. (2013). Low-viscosity hydroxypropylcellulose (HPC) grades SL and SSL: Versatile pharmaceutical polymers for dissolution enhancement, controlled release, and pharmaceutical processing. Aaps Pharmscitech.

[43] Aho J., Van Renterghem J., Arnfast L., De Beer T., Rantanen J. (2017). The flow properties and presence of crystals in drug-polymer mixtures: Rheological investigation combined with light microscopy. Int. J. Pharm..

[44] Censi R., Gigliobianco M.R., Casadidio C., Di Martino P. (2018). Hot melt extrusion: Highlighting physicochemical factors to be investigated while designing and optimizing a hot melt extrusion process. Pharmaceutics.

[45] Uekama K., Ikegami K., Wang Z., Horiuchi Y., Hirayama F. (1992). Inhibitory effect of 2-hydroxypropyl-β-cyclodextrin on crystal-growth of nifedipine during storage: Superior dissolution and oral bioavailability compared with polyvinylpyrrolidone K-30. J. Pharm. Pharmacol..

[46] Baird J.A., Van Eerdenbrugh B., Taylor L.S. (2010). A classification system to assess the crystallization tendency of organic molecules from undercooled melts. J. Pharm. Sci..

[47] Buyukgoz G.G., Soffer D., Defendre J., Pizzano G.M., Davé R.N. (2020). Exploring Tablet Design Options for Tailoring Drug Release and Dose via Fused Deposition Modeling (FDM) 3D Printing. Int. J. Pharm..

[48] Cetindag E., Pentangelo J., Cespedes T.A., Davé R.N. (2020). Effect of solvents and cellulosic polymers on quality attributes of films loaded with a poorly water-soluble drug. Carbohydr. Polym..

[49] Griffin S.R., Takanti N., Sarkar S., Song Z., Vogt A.D., Danzer G.D., Simpson G.J. (2020). Disparities of Single-Particle Growth Rates in Buried Versus Exposed Ritonavir Crystals within Amorphous Solid Dispersions. Mol. Pharm..

[50] Brenken B., Favaloro A., Barocio E., DeNardo N.M., Pipes R.B. Development of a model to predict temperature history and crystallization behavior of 3D printed parts made from fiber-reinforced thermoplastic polymers. Proceedings of the SAMPE Conference Proceeding.

[51] Sun D.D., Wen H., Taylor L.S. (2016). Non-sink dissolution conditions for predicting product quality and in vivo performance of supersaturating drug delivery systems. J. Pharm. Sci..

[52] Goyanes A., Martinez P.R., Buanz A., Basit A.W., Gaisford S. (2015). Effect of geometry on drug release from 3D printed tablets. Int. J. Pharm..

[53] Sadia M., Arafat B., Ahmed W., Forbes R.T., Alhnan M.A. (2018). Channelled tablets: An innovative approach to accelerating drug release from 3D printed tablets. J. Control. Release.

[54] Rahman M., Coelho A., Tarabokija J., Ahmad S., Radgman K., Bilgili E. (2020). Synergistic and Antagonistic Effects of Various Amphiphilic Polymer Combinations in Enhancing Griseofulvin Release from Ternary Amorphous Solid Dispersions. Eur. J. Pharm. Sci..

[55] Öblom H., Zhang J., Pimparade M., Speer I., Preis M., Repka M., Sandler N. (2019). 3D-printed isoniazid tablets for the treatment and prevention of tuberculosis—Personalized dosing and drug release. AAPS PharmSciTech.

[56] Pietrzak K., Isreb A., Alhnan M.A. (2015). A flexible-dose dispenser for immediate and extended release 3D printed tablets. Eur. J. Pharm. Biopharm..

[57] Li M., Ioannidis N., Gogos C., Bilgili E. (2017). A comparative assessment of nanocomposites vs. amorphous solid dispersions prepared via nanoextrusion for drug dissolution enhancement. Eur. J. Pharm. Biopharm..

[58] Bühler V. (2007). Kollicoat Grades: Functional Polymers for the Pharmaceutical Industry.

[59] Callister W.D. (2007). Materials Science and Engineering: An Introduction.

[60] Rumondor A.C., Marsac P.J., Stanford L.A., Taylor L.S. (2009). Phase behavior of poly (vinylpyrrolidone) containing amorphous solid dispersions in the presence of moisture. Mol. Pharm..

[61] Goyanes A., Allahham N., Trenfield S.J., Stoyanov E., Gaisford S., Basit A.W. (2019). Direct powder extrusion 3D printing: Fabrication of drug products using a novel single-step process. Int. J. Pharm..

[62] Rahman M., Arevalo F., Coelho A., Bilgili E. (2019). Hybrid nanocrystal–amorphous solid dispersions (HyNASDs) as alternative to ASDs for enhanced release of BCS Class II drugs. Eur. J. Pharm. Biopharm..

[63] Purohit H.S., Taylor L.S. (2017). Phase behavior of ritonavir amorphous solid dispersions during hydration and dissolution. Pharm. Res..

[64] Rahman M., Ahmad S., Tarabokija J., Bilgili E. (2020). Roles of surfactant and polymer in drug release from spray-dried hybrid nanocrystal-amorphous solid dispersions (HyNASDs). Powder Technol..

[65] Bhakay A., Azad M., Bilgili E., Dave R. (2014). Redispersible fast dissolving nanocomposite microparticles of poorly water-soluble drugs. Int. J. Pharm..

[66] Thommes M., Ely D.R., Carvajal M.T., Pinal R. (2011). Improvement of the dissolution rate of poorly soluble drugs by solid crystal suspensions. Mol. Pharm.

[67] Tagami T., Nagata N., Hayashi N., Ogawa E., Fukushige K., Sakai N., Ozeki T. (2018). Defined drug release from 3D-printed composite tablets consisting of drug-loaded polyvinylalcohol and a water-soluble or water-insoluble polymer filler. Int. J. Pharm..

[68] Li Q., Wen H., Jia D., Guan X., Pan H., Yang Y., Yu S., Zhu Z., Xiang R., Pan W. (2017). Preparation and investigation of controlled-release glipizide novel oral device with three-dimensional printing. Int. J. Pharm..

[69] Ritger P.L., Peppas N.A. (1987). A simple equation for description of solute release II. Fickian and anomalous release from swellable devices. J. Control. Release.

[70] Alhijjaj M., Nasereddin J., Belton P., Qi S. (2019). Impact of Processing Parameters on the Quality of Pharmaceutical Solid Dosage Forms Produced by Fused Deposition Modeling (FDM). Pharmaceutics.

[71] Korte C., Quodbach J. (2018). Formulation development and process analysis of drug-loaded filaments manufactured via hot-melt extrusion for 3D-printing of medicines. Pharm. Dev. Technol..

[72] Verstraete G., Samaro A., Grymonpré W., Vanhoorne V., Van Snick B., Boone M., Hellemans T., Van Hoorebeke L., Remon J.P., Vervaet C. (2018). 3D printing of high drug loaded dosage forms using thermoplastic polyurethanes. Int. J. Pharm..

[73] Bennett R.C., Keen J.M., Bi Y., Porter S., Dürig T., McGinity J.W. (2015). Investigation of the interactions of enteric and hydrophilic polymers to enhance dissolution of griseofulvin following hot melt extrusion processing. J. Pharm. Pharmacol..

[74] Yadav V., Yadav A. (2009). Effect of different stabilizers and polymers on spherical agglomerates of gresiofulvine by emulsion solvent diffusion (ESD) system. Int. J. Pharm. Tech. Res.

[75] Sarode A.L., Malekar S.A., Cote C., Worthen D.R. (2014). Hydroxypropyl cellulose stabilizes amorphous solid dispersions of the poorly water soluble drug felodipine. Carbohydr. Polym..

[76] Sathigari S.K., Radhakrishnan V.K., Davis V.A., Parsons D.L., Babu R.J. (2012). Amorphous-state characterization of efavirenz—polymer hot-melt extrusion systems for dissolution enhancement. J. Pharm. Sci..

[77] Al-Obaidi H., Buckton G. (2009). Evaluation of griseofulvin binary and ternary solid dispersions with HPMCAS. AAPS PharmSciTech.

[78] Żarów A., Zhou B., Wang X., Pinal R., Iqbal Z. (2011). Spectroscopic and X-ray diffraction study of structural disorder in cryomilled and amorphous griseofulvin. Appl. Spectrosc..

[79] Gogos C.G., Liu H., Wang P. (2012). Laminar Dispersive and Distributive Mixing with Dissolution and Applications to Hot-Melt Extrusion. Hot Melt Extrus. Pharm. Appl..

[80] Tidau M., Kwade A., Finke J.H. (2019). Influence of High, Disperse API Load on Properties along the Fused-Layer Modeling Process Chain of Solid Dosage Forms. Pharmaceutics.

[81] Loftsson T., Fri H., Gu T.K. (1996). The effect of water-soluble polymers on aqueous solubility of drugs. Int. J. Pharm..

[82] Goyanes A., Buanz A.B., Hatton G.B., Gaisford S., Basit A.W. (2015). 3D printing of modified-release aminosalicylate (4-ASA and 5-ASA) tablets. Eur. J. Pharm. Biopharm..

[83] Sun D.D., Lee P.I. (2015). Probing the mechanisms of drug release from amorphous solid dispersions in medium-soluble and medium-insoluble carriers. J. Control. Release.

[84] Augustijns P., Brewster M.E. (2012). Supersaturating drug delivery systems: Fast is not necessarily good enough. J. Pharm. Sci..

[85] Mendyk A., Pacławski A., Szlek J., Jachowicz R. (2013). PhEq_bootstrap: Open-source software for the simulation of f2 distribution in cases of large variability in dissolution profiles. Dissolution Technol..

[86] Paixão P., Gouveia L.F., Silva N., Morais J.A. (2017). Evaluation of dissolution profile similarity–Comparison between the f2, the multivariate statistical distance and the f2 bootstrapping methods. Eur. J. Pharm. Biopharm..

