# Supplementary material for: Enhanced Supersaturation via Fusion-Assisted Amorphization during FDM 3D Printing of Crystalline Poorly Soluble Drug Loaded Filaments
Source: Pharmaceutics. 2021 Nov 4;13(11):1857. doi: 10.3390/pharmaceutics13111857 (PMC8618474; doi:10.3390/pharmaceutics13111857)
Supplement: Supplementary file 1 [file pharmaceutics-13-01857-s001.zip › pharmaceutics-1418038-supplementary.pdf]

Supplementary Material

Enhanced Supersaturation via Fusion-assisted Amorphization during FDM 3D Printing of Crystalline Poorly Soluble Drug Loaded Filaments

Guluzar Gorkem Buyukgoz [1], Christopher Gordon Kossor [1], Rajesh N. Davé [1,*],

[1] *Otto H. York Department of Chemical and Materials Engineering,*

New Jersey Institute of Technology, Newark, New Jersey, USA

**Correspondence to* Rajesh N. Davé, (Phone:+1-973-596-5860. E-mail address: dave@njit.edu)

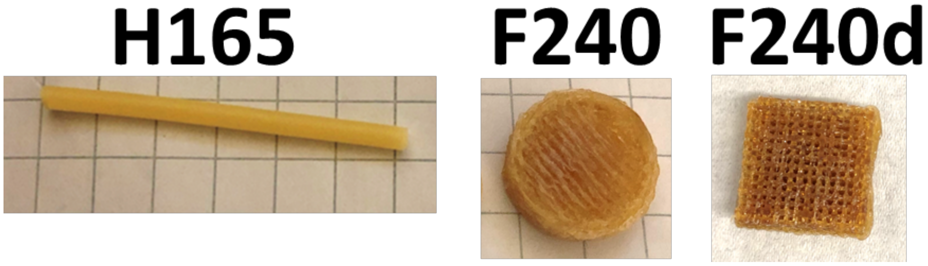

Figure S1: Digital images of the filament, H165 (left), and FDM 3D printed tablets for cases F240 (middle) and F240d (right).

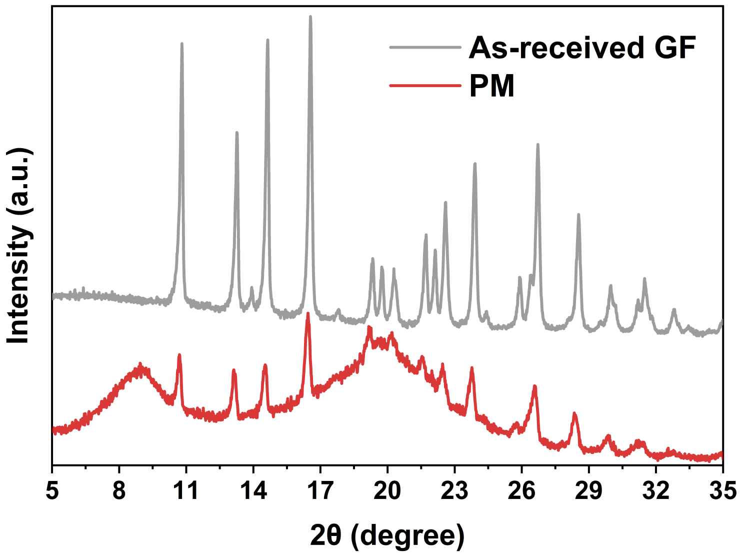

Figure S2: XRPD pattern of as-received GF and physical mixture (PM).

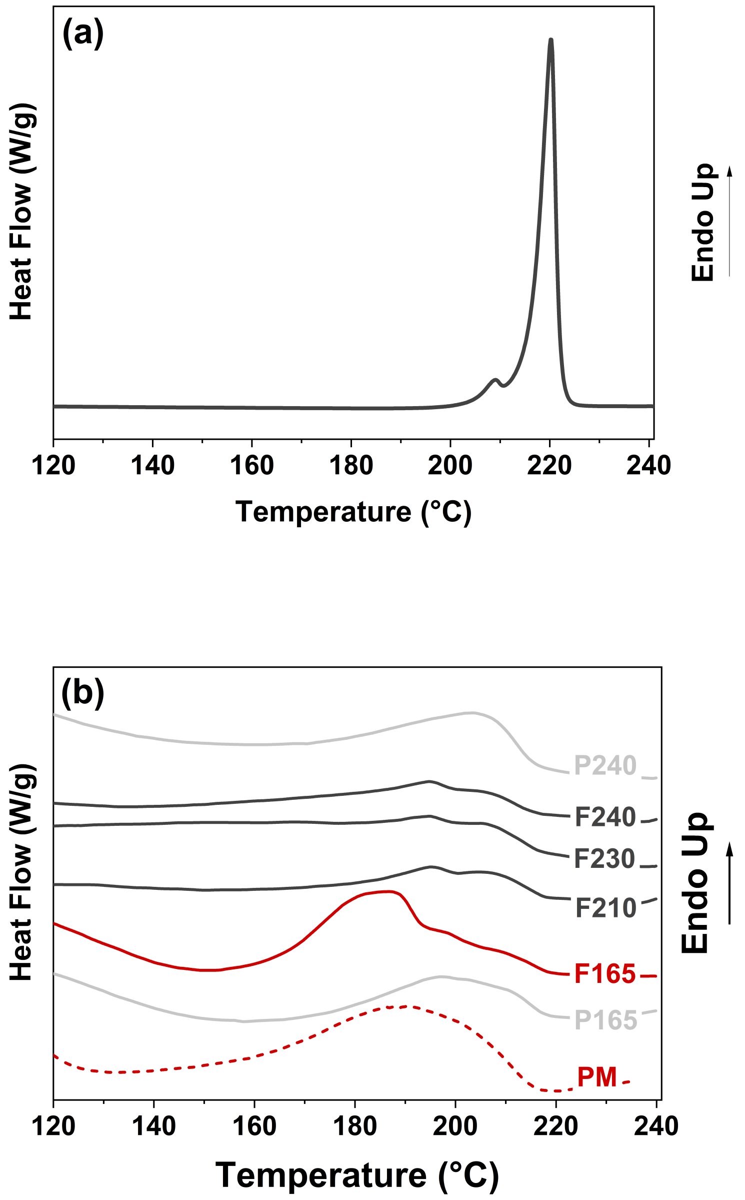

Figure S3. DSC thermograms of; (a) as-received GF, (b) physical mixture (PM), placebo (P165 and P240), and GF loaded (F165-F240) printed tablets.

In DSC thermograms, Figure S1, GF powder showed a melting endotherm at 220.4 ºC. Although HPC and KP are XRD amorphous [1,2], the endotherms in the range of 166–218 ºC could be attributed melting of their small crystalline domains [3]. Due to the overlapping thermal events in the similar temperature range, the PM showed only a broad endotherm around 130–217 ºC, which prevented the

calculation of GF crystallinity. The melting peak of F165 appeared at 186.6 ºC along with two minor endothermic shoulders around 195–201 and 205–218 ºC. HPC has been reported to cause melting point depression due to its high degree of amorphous content [3,4]. Thus, the peak at 186.6 ºC was considered as the melting event of GF [2]. Lacking that peak in the thermogram of the placebo tablet, P165, further supported the outcome. For H165, these results indicated the presence of GF crystals in the amorphous HPC-KP. As the processing temperature increased through F210–240, only two endothermic shoulders appearedaround 195 ºC and 206, ºC, and the GF peak at 186 ºC was disappeared, Figure S1. That indicated GF molecularly dispersed in the formulation. Interestingly, such formation of ASDs could be attributed to the temperature-induced miscibility [5] for GF-HPC-KP formulation. Although the extent of amorphization could not be calculated with DSC, it was supported with FT-IR results (see Section 3.3). Similar behavior was observed by [6,7].

Figure S4. Microscopy images of 3D tablets printed at 165 °C and 240 °C.

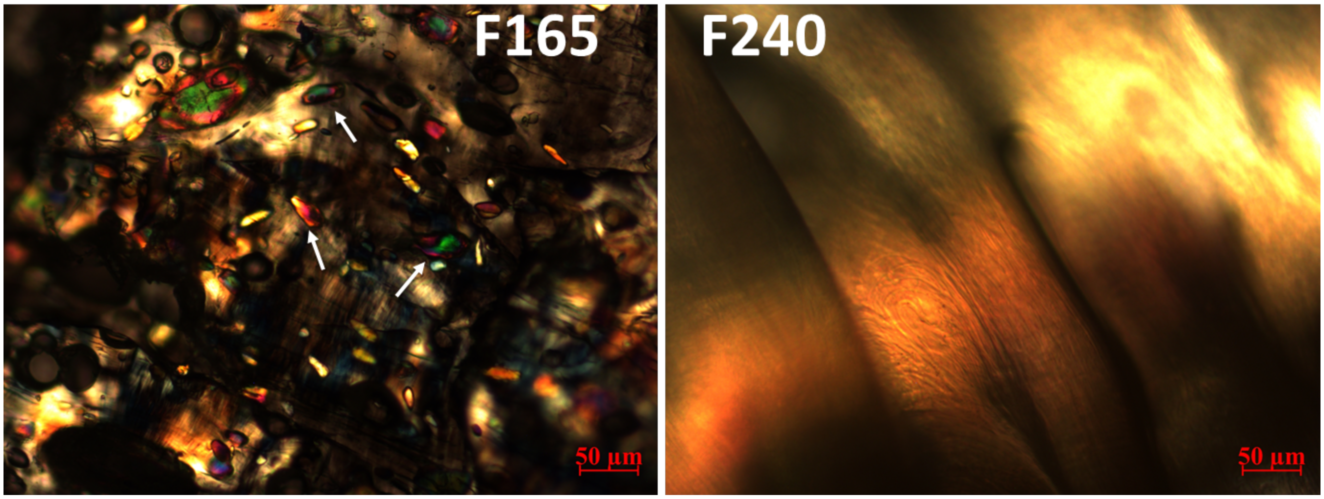

Table S1. Extent of GF supersaturation for various tablet designs along with the fitting parameters of the corresponding fitting dissolution curves.

Run	*Extent of Supersaturation (%)	Fitting Parameters		
		k (%/min^n)	n	R^2
F210	61.8	-	-	-
F230	95.7	-	-	-
F240	153.3	0.16	0.94	0.988
F240a	246.8	5.07	0.46	0.975
F240b	214.9	1.86	0.58	0.988
F240c	225.6	1.71	0.59	0.977
F240d	293.0	7.13	0.38	0.918

*The values for extent of GF supersaturation are calculated from the dissolution curves at the time periods between 9–12h.

Table S2. 3D printed tablets along with their corresponding tablet masses and theoretical drug amounts.

Tablet Design	Tablet Mass (mg)	Theoretical Drug Amount (mg)
F240	624.95±1.50	98.57±0.24
F240a	569.35±46.62	89.80±7.35
F240b[1]	598.33±20.17	91.66±3.09
F240c	573.33±41.10	90.43±6.48
F240d	612.33 ±24.86	96.58±3.92

[1]The total tablet mass and drug amount of 33 mini-tablets.

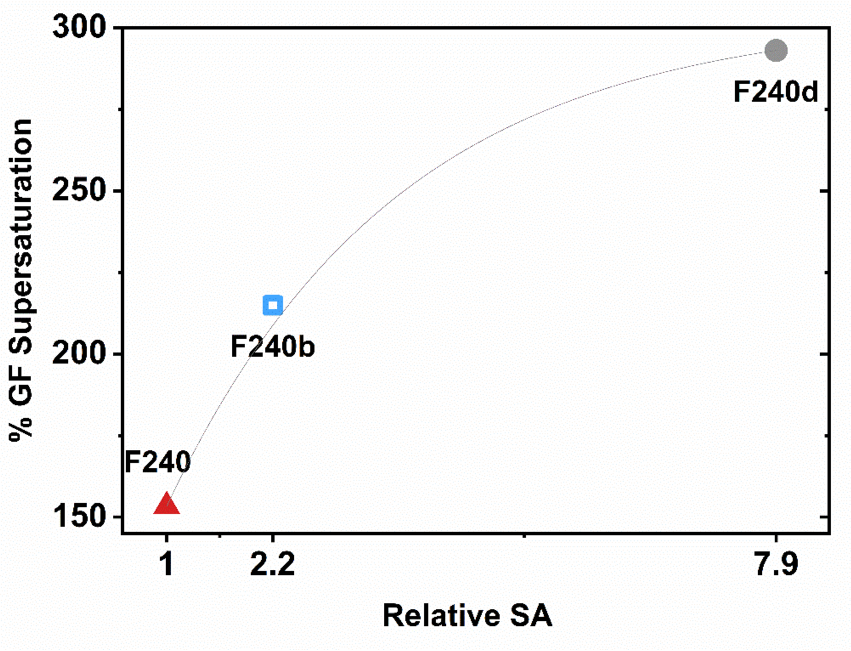

Figure S5. Relative surface areas (SA) of 3D tablets (F240, F240b and F240d) and corresponding level of GF supersaturation. The trendline depicts monotonously increasing % GF supersaturation as a function of relative SA.

References

1. Petry, I.; Löbmann, K.; Grohganz, H.; Rades, T.; Leopold, C.S. Solid state properties and drug release behavior of co-amorphous indomethacin-arginine tablets coated with Kollicoat® Protect. *Eur. J. Pharm. Biopharm.* **2017**, *119*, 150-160.
2. Rahman, M.; Coelho, A.; Tarabokija, J.; Ahmad, S.; Radgman, K.; Bilgili, E. Synergistic and Antagonistic Effects of Various Amphiphilic Polymer Combinations in Enhancing Griseofulvin Release from Ternary Amorphous Solid Dispersions. *Eur. J. Pharm. Sci.* **2020**, 105354.
3. Sarode, A.; Wang, P.; Cote, C.; Worthen, D.R. Low-viscosity hydroxypropylcellulose (HPC) grades SL and SSL: versatile pharmaceutical polymers for dissolution enhancement, controlled release, and pharmaceutical processing. *Aaps Pharmscitech* **2013**, *14*, 151-159.
4. DiNunzio, J.C.; Brough, C.; Hughey, J.R.; Miller, D.A.; Williams III, R.O.; McGinity, J.W. Fusion production of solid dispersions containing a heat-sensitive active ingredient by hot melt extrusion and Kinetisol® dispersing. *Eur. J. Pharm. Biopharm.* **2010**, *74*, 340-351.
5. Gogos, C.G.; Liu, H.; Wang, P. Laminar Dispersive and Distributive Mixing with Dissolution and Applications to Hot-Melt Extrusion. *Hot-Melt Extrusion: Pharmaceutical Applications* **2012**, 261-284.
6. Vasanthavada, M.; Tong, W.-Q.T.; Joshi, Y.; Kislalioglu, M.S. Phase behavior of amorphous molecular dispersions II: Role of hydrogen bonding in solid solubility and phase separation kinetics. *Pharm. Res.* **2005**, *22*, 440-448.
7. Lu, Q.; Zografi, G. Phase behavior of binary and ternary amorphous mixtures containing indomethacin, citric acid, and PVP. *Pharm. Res.* **1998**, *15*, 1202-1206.
